# Identifying Blood Transcriptome Biomarkers of Alzheimer’s Disease Using Transgenic Mice

**DOI:** 10.1007/s12035-020-02058-2

**Published:** 2020-08-20

**Authors:** Shinichiro Ochi, Jun-ichi Iga, Yu Funahashi, Yuta Yoshino, Kiyohiro Yamazaki, Hiroshi Kumon, Hiroaki Mori, Yuki Ozaki, Takaaki Mori, Shu-ichi Ueno

**Affiliations:** Department of Neuropsychiatry, Molecules and Function, Ehime University Graduate School of Medicine, Shitsukawa, Toon, Ehime 791-0295 Japan

**Keywords:** Blood, Transcriptome, Biomarker, Hippocampus, 3xTg-AD mice

## Abstract

The testing of pathological biomarkers of Alzheimer’s disease (AD), such as amyloid beta and tau, is time-consuming, expensive, and invasive. Here, we used 3xTg-AD mice to identify and validate putative novel blood transcriptome biomarkers of AD that can potentially be identified in the blood of patients. mRNA was extracted from the blood and hippocampus of 3xTg-AD and control mice at different ages and used for microarray analysis. Network and functional analyses revealed that the differentially expressed genes between AD and control mice modulated the immune and neuroinflammation systems. Five novel gene transcripts (*Cdkn2a*, *Apobec3*, *Magi2*, *Parp3*, and *Cass4*) showed significant increases with age, and their expression in the blood was collated with that in the hippocampus only in AD mice. We further assessed previously identified candidate biomarker genes. The expression of *Trem1* and *Trem2* in both the blood and brain was significantly increased with age. Decreased *Tomm40* and increased *Pink1* mRNA levels were observed in the mouse blood. The changes in the expression of *Snca* and *Apoe mRNA* in the mouse blood and brain were similar to those found in human AD blood. Our results demonstrated that the immune and neuroinflammatory system is involved in the pathophysiologies of aging and AD and that the blood transcriptome might be useful as a biomarker of AD.

**Electronic supplementary material:**

The online version of this article (10.1007/s12035-020-02058-2) contains supplementary material, which is available to authorized users.

## Introduction

Currently, the diagnosis of Alzheimer’s disease (AD) is mainly based on the assessment of clinical symptoms and cognitive tests. Several tools are used for the diagnosis of AD, and these include the identification of biomarkers from brain scans and lumbar puncture procedures. The levels of amyloid beta and tau proteins in the cerebrospinal fluid and those detected by positron emission tomography are highly accurate markers for the detection of AD pathology in the brain. However, the assessment of these biomarkers is invasive and time- and cost-consuming [1]. Thus, researchers are aiming to develop simple blood tests that can accurately diagnose the disease and potentially predict its prodromal stage prior to the appearance of clinical symptoms [2].

In addition to the presence of amyloid plaques in the brain parenchyma and intraneuronal neurofibrillary tangles, emerging evidence suggests the existence of additional AD pathophysiological pathways, such as innate immune responses, neuroinflammation, and vascular and cell membrane dysregulation [3–5]. Our previous studies suggest that the detection of transcriptome biomarkers related to cell stress and inflammation in the peripheral blood has significant potential as a minimally invasive and inexpensive diagnostic tool for the diagnosis and early detection of developing AD [6–15]. Although DNA methylation and RNA expression changes in blood might have utility as biomarkers of cognitive dysfunction and brain aging [16, 17], the major limitation of blood biomarkers of AD is the lack of a direct correlation with biomarkers in brain tissues. Thus, we used AD model mice to identify genes that exhibit similar changes in the blood and hippocampus. If these genes are implicated in the pathophysiology of AD, they might be good candidate blood transcriptome biomarkers of AD.

In the present study, we simultaneously collected brain and blood samples from 3xTg-AD mice to identify novel blood transcriptome biomarkers of AD and to validate our previous biomarkers.

## Materials and Methods

### Animal Models

Male 3xTg-AD mice (*n* = 8 each at 12 and 52 weeks of age (w.o.a.)) and age-matched male B6129SF2 WT mice (*n* = 8 each at 12 and 52 w.o.a.) were bred at Ehime University from parents originally purchased from Jackson Laboratory (3xTg-AD, MMRRC #34830; B6129SF1/J, JAX #101043). For validation analysis, additional male 3xTg-AD mice and age-matched male B6129SF2 WT mice (*n* = 8 each at 36 w.o.a.) were also bred under the same conditions. All the mice were housed in a specific pathogen-free facility with climate-controlled conditions consisting of room temperature (22 ± 2 °C), 50% humidity. and a 12-h light/12-h dark cycle. The mice were provided with water and a standard diet (Oriental Yeast Co., Ltd.) at libitum. At 12, 36, or 52 w.o.a., the mice were anesthetized and killed by decapitation, and 12, 36, and 52 weeks were selected as representative young, middle, and old ages based on previous studies that examined the course of pathological and behavioral changes in 3xTg AD mice [18, 19]. The hippocampi were removed and immediately stored, and blood was collected using RNeasy Protect Animal Blood Tubes (QIAGEN, #73224) and stored at − 80 °C until RNA processing. The sample size (*n* = 8 each) was the minimum number necessary and allowed by the ethical committee. Most of the AD and control mice did not die before reaching 52 w.o.a. The animal experiments were approved by the Animal Experiment Committee of Ehime University (#28-25) and were performed in accordance with the Guidelines for Animal Experiments at Ehime University.

### Microarray Analysis

Total RNA was extracted from frozen hippocampi using the RNeasy Mini Kit (QIAGEN, #74104) and from blood using the RNeasy Protect Animal Blood Kit (QIAGEN, #73224) according to the manufacturer’s instructions. The RNA concentrations were determined using a NanoDrop1000 instrument (Thermo Fisher Scientific), and its quality was assessed by determining the RNA integrity number using an Agilent 2100 Bioanalyzer (Agilent). All the samples used for microarray analysis met the following conditions: A260/A280 ≥ 1.8, A260/A230 ≥ 2.0, and RNA integrity number ≥ 7.0. Using 50 ng of RNA, amplified and biotinylated sense-strand DNA targets were generated using the Low Input Quick Amp Labeling Kit (Agilent). The hybridization, washing, and scanning steps were conducted using the Gene Expression Hybridization Kit (Agilent), Gene Expression Wash Pack (Agilent, #5188–5327), and Agilent Microarray Scanner (G2505C) with SurePrint G3 Mouse 8x60K version 2.0. The 75th percentile shift normalization and baseline transformation (baseline to median of all the samples (all the blood samples or all the hippocampal samples)) were performed using Agilent GeneSpring GX version 14.9. To determine the independent and combined effects of the AD mouse model and aging, comparisons among groups (AD12 Hip (AD mouse hippocampus at 12 w.o.a.), AD52 Hip (AD mouse hippocampus at 52 w.o.a), C12 Hip (control mouse hippocampus at 12 w.o.a), and C52 Hip (control mouse hippocampus at 52 w.o.a.)) or AD12 bld )(AD mouse blood at 12 w.o.a), AD52 bld (AD mouse blood at 52 w.o.a.), C12 bld (control mouse blood at 12 w.o.a), and C52 bld (control mouse blood at 52 w.o.a)) were performed with two-way analysis of variance (ANOVA) followed by Tukey’s honestly significant difference (HSD) test (two-way ANOVA: *P* < 0.1, fold change ≥ 1.5 or ≤ − 1.5, Tukey’s HSD: *P* < 0.05). The network and functional analyses were performed using IPA (QIAGEN) (https://www.qiagenbioinformatics.com/products/ingenuity-pathway-analysis) [20]. All microarray data were deposited in the GEO database (accession number GSE144459).

### PCR

For the validation of microarray data, the expression levels of various mRNAs were measured by quantitative reverse transcription-PCR (qPCR) using the StepOnePlus Real-Time PCR System (Applied Biosystems). The TaqMan probes used in this study were Mm00494449_m1 for mouse *Cdkn2a* and Mm99999915_g1 for mouse *Gapdh* (Applied Biosystems). RT-PCR was conducted using the TaqMan gene expression master mix with a final volume of 10 μL. The expression levels were measured in duplicate using the ΔΔCt method. We included the same sample in all the plates as a calibrator to adjust for differences among the plates.

### Validation Analyses Using Public Functional Genomics Data

To validate biomarkers in postmortem human brain and other models of Alzheimer disease (APP_PS1 model mouse), we used the expression data of Gene Expression Omnibus database (GSE48350) [21] and (GSE111737) [22], respectively. From human hippocampal data (GSE48350), young male controls (*N* = 9, mean age 30.6 ± 11.0), old male controls (*N* = 10, 85.8 ± 6.8), and male patients with Alzheimer’s disease (N = 9, 84.1 ± 6.9) were examined, which correspond to “C12 Hip vs. C52 Hip,” “C12 Hip vs. AD52 Hip,” and “C52 Hip vs. AD52 Hip” in our data. From APP_PS1 mouse hippocampal data (GSE111737), wild-type male mice (*n* = 6, 8 months old) and APP_PS1 male mice (*n* = 7, 8 months old) were examined, which correspond to “C52 Hip vs. AD52 Hip” in our data.

## Results

### Differential Gene Expression Between Male 3xTg-AD Mice and Age-Matched Male B6129SF2 WT Mice

The analysis of gene expression revealed positive correlations among the same tissues from the mice belonging to the same group, such as between the AD12 and AD52 hip samples, whereas negative correlations were found among the same tissues from mice belonging to different groups, such as between the AD and C hip samples. No correlations were consistently observed among different tissues, such as between the AD blood samples and the AD hip samples (Supplementary Figure 1).

We selected differentially expressed genes (DEGs) in AD mice that met the following criteria: fold change (≥ 1.5 or ≤ −1.5) and significance level (Tukey’s HSD: *P* < 0.05). This analysis showed that 128 and 251 were significantly increased and significantly decreased, respectively, only in the AD mouse blood. In contrast, 196 and genes were significantly increased and significantly decreased, respectively, only in the AD mouse hippocampus (Supplementary Table 1).

### Network and Functional Analyses

To determine the functions of DEGs, network and functional analyses were conducted using IPA (Tables 1 and 2). The network analyses of the top canonical pathways showed that DEGs that were only found in the AD mouse blood were involved in “3-phosphoinositide degradation,” “primary immunodeficiency signaling,” and “D-myo-inositol-5-phosphate metabolism.” The top upstream regulators included IL3, IL21 (inhibited), IL15RA, IFNA2 (inhibited), and TLN1. In the causal network, MAP3K13, TLR3, and androgen-AR were predicted to be inhibited, whereas KAT7 was predicted to be activated. The analysis of the top diseases and biological functions revealed that “cancer,” “cellular development,” and “hematological system development and function” showed the most substantial enrichment.

**Table 1:** Network and functional analyses of differentially expressed genes found only in the AD mouse blood

**Top canonical pathways**			
**Name**		***P*** **value**	**Overlap**
1	3-phosphoinositide degradation	1.12E-03	6.2% 9/146
2	Primary immunodeficiency signaling	3.18E-03	11.4% 4/35
3	D-myo-inositol-5-phosphate metabolism	4.49E-03	5.4% 8/147
4	Superpathway of inositol phosphate compounds	6.11E-03	4.8% 9/188
5	3-Phosphoinositide biosynthesis	6.38E-03	5.1% 8/156
**Top upstream regulators**			
**Name**		***P*** **value**	**Predicted activation**
1	IL3	3.85E-07	
2	IL21	1.38E-05	Inhibited
3	IL15RA	1.69E-05	
4	IFNA2	4.21E-05	Inhibited
5	TLN1	5.55E-05	
**Causal network**			
**Name**		***P*** **value**	**Predicted activation**
1	MAP3K13	4.93E-07	Inhibited
2	Interferon beta-1a	5.66E-07	
3	TLR7	6.02E-07	Inhibited
4	KAT7	7.48E-07	Activated
5	Androgen-AR	2.10E-06	Inhibited
**Top diseases and bio functions**			
**Diseases and disorders**			
**Name**		***P*** **value range**	**Number of molecules**
1	Cancer	9.16E-03–5.36E-06	287
2	Hematological disease	8.80E-03–5.36E-06	106
3	Immunological disease	8.80E-03–5.36E-06	131
4	Organismal injury and abnormalities	9.27E-03–5.36E-06	291
5	Inflammatory response	6.92E-03–2.02E-05	46
**Molecular and cellular functions**			
**Name**		***P*** **value range**	**Number of molecules**
1	Cellular development	9.27E-03–5.57E-07	111
2	Cellular growth and proliferation	9.27E-03–5.57E-07	113
3	Cell death and survival	9.18E-03–4.80E-06	117
4	Cell cycle	9.18E-03–4.80E-06	63
5	Cell-to-cell signaling and interaction	9.27E-03–2.02E-05	59
**Physiological system development and function**			
**Name**		***P*** **value range**	**Number of molecules**
1	Hematological system development and function	9.27E-03–4.13E-08	84
2	Hematopoiesis	9.27E-03–4.13E-08	54
3	Tissue development	9.27E-03–5.57E-07	75
4	Embryonic development	9.27E-03–6.04E-06	57
5	Lymphoid tissue structure and development	9.27E-03–6.04E-06	60

**Table 2:** Network and functional analyses of differentially expressed genes found only in the AD mouse hippocampus

**Top canonical pathways**			
**Name**		***P*** **value**	**Overlap**
1	Dendritic cell maturation	1.63E-05	6.6% 10/151
2	Altered T cell and B cell signaling in rheumatoid arthritis	4.56E-05	9.0% 7/78
3	Hepatic fibrosis/hepatic stellate cell activation	6.99E-05	5.6% 10/179
4	Apelin liver signaling pathway	2.59E-04	15.4% 4/26
5	Communication between innate and adaptive immune cells	1.25E-03	7.6% 5/66
**Top upstream regulators**			
**Name**		***P*** **value**	**Predicted activation**
1	L2HGDH	3.17E-10	
2	Lipopolysaccharide	9.44E-08	Activated
3	TBX5	3.08E-07	
4	FOSL1	5.73E-07	
5	CR1L	9.23E-07	
**Causal network**			
**Name**		***P*** **value**	**Predicted activation**
1	L2HGDH	3.17E-10	
2	Icilin	4.74E-07	
3	LY96	4.79E-07	Activated
4	D-allose	7.21E-07	Inhibited
5	CR1L	9.23E-07	
**Top diseases and bio functions**			
**Diseases and disorders**			
**Name**		***P*** **value range**	**Number of molecules**
1	Endocrine system disorders	2.97E-03–1.71E-08	77
2	Gastrointestinal disease	3.09E-03–1.71E-08	105
3	Metabolic disease	1.44E-03–1.71E-08	51
4	Organismal injury and abnormalities	3.23E-03–1.71E-08	163
5	Immunological disease	2.97E-03–6.72E-08	72
**Molecular and cellular functions**			
**Name**		***P*** **value range**	**Number of molecules**
1	Cell-to-cell signaling and interaction	2.97E-03–3.13E-09	54
2	Cellular movement	3.17E-03–3.13E-09	62
3	Cell death and survival	3.34E-03–8.74E-08	36
4	Cellular development	2.98E-03–2.93E-06	53
5	Cellular growth and proliferation	2.98E-03–2.93E-06	49
**Physiological system development and function**			
**Name**		***P*** **value range**	**Number of molecules**
1	Hematological system development and function	3.09E-03–3.13E-09	57
2	Immune cell trafficking	3.09E-03–3.13E-09	42
3	Lymphoid tissue structure and development	2.97E-03–2.93E-06	48
4	Cell-mediated immune response	2.49E-03–1.11E-05	28
5	Connective tissue development and function	2.98E-03–1.66E-05	26

In contrast, the canonical pathway analysis revealed that the DEGs that were only found in the AD mouse hippocampus were involved in “dendritic cell maturation,” “altered T cell and B cell signaling in rheumatoid arthritis,” and “communication between innate and adaptive immune cells.” The top upstream regulators included L2HGDH, lipopolysaccharide (activated), TBX5, FOSL1, and CR1L. In the causal network, L2HGDH, icilin, LY96 (activated), D-allose (inhibited), and CR1L showed the most substantial enrichment. The analysis of the top diseases and biological functions showed that “endocrine system disorders,” “cell-to-cell signaling and interaction,” and “hematological system development and function” exhibited the most substantial enrichment.

### Blood Transcriptome Biomarkers of AD

To identify novel blood transcriptome biomarkers of AD, we identified genes that showed significant changes with age and that exhibited correlations between the blood and hippocampus only in AD mice (Table 3). Five genes (*Cdkn2a*, *Apobec3*, *Magi2*, *Parp3*, and *Cass4*) showed significant increases in expression with increasing age, and their expression in the blood was correlated with that in the hippocampus only in AD mice.

**Table 3:** Genes that showed significant changes in expression with age and that exhibited correlations between the blood and hippocampus only in AD mice (C12, control mice at 12 w.o.a.; C52, control mice at 52 w.o.a.; AD12, AD mice at 12 w.o.a.; AD52, AD mice at 52 w.o.a.; bld, blood; hip, hippocampus; FC, fold change; w.o.a., weeks of age). The significantly altered genes (two-way ANOVA: *P* < 0.1, fold change ≥ 1.5 or ≤ − 1.5, Tukey’s HSD: *P* < 0.05) are indicated in italics

**Gene symbol**	**p (Mouse-Age)**	**p (Mouse)**	**p (Age)**	**C12 bld vs. C52 bld**		**C12 bld vs. AD12 bld**		**C12 bld vs. AD52 bld**		**C52 bld vs. AD12 bld**		**C52 bld vs. AD52 bld**		**AD12 bld vs. AD52 bld**	
				**p**	**FC**	**p**	**FC**	**p**	**FC**	**p**	**FC**	**p**	**FC**		**FC**
*Cdkn2a*	*0.0570*	0.1532	0.1590	0.9809	− 1.1766	0.9830	− 1.1691	0.1906	*2.4127*	1.0000	1.0064	*0.0922*	*2.8389*	*0.0950*	*2.8208*
*Apobec3*	0.1030	0.7599	*0.0970*	1.0000	1.0041	0.7655	− 1.1946	0.4907	1.2990	0.7531	− 1.1995	0.5039	1.2937	*0.0993*	*1.5518*
*Magi2*	0.7870	0.2662	*0.0849*	0.7101	1.4178	0.9284	1.2198	0.1893	*1.9613*	0.9669	− 1.1623	0.7535	1.3834	0.4766	*1.6079*
*Parp3*	*0.0421*	0.8478	*0.0578*	0.9996	− 1.0200	0.5284	− 1.2876	0.4306	1.3279	0.5940	− 1.2624	0.3720	1.3544	*0.0339*	*1.7098*
*Cass4*	*0.0971*	*0.0001*	*0.0012*	0.5507	1.1953	0.2224	1.3022	*0.0002*	*2.1544*	0.9182	1.0895	*0.0009*	*1.8023*	*0.0043*	*1.6544*
**Gene symbol**	**p (Mouse-Age)**	**p (Mouse)**	**p (Age)**	**C12 Hip vs. C52 Hip**		**C12 Hip vs. AD12 Hip**		**C12 Hip vs. AD52 Hip**		**C52 Hip vs. AD12 Hip**		**C52 Hip vs. AD52 Hip**		**AD12 Hip vs. AD52 Hip**	
				**P**	**FC**	**p**	**FC**	**p**	**FC**	**p**	**FC**	**p**	**FC**	**p**	**FC**
*Cdkn2a*	*0.0544*	*0.0111*	*0.0000*	0.1687	*1.7901*	*0.0120*	*− 2.4951*	0.3816	*1.5594*	*0.0002*	*− 4.4663*	0.9574	− 1.1479	*0.0003*	*3.8909*
*Apobec3*	*0.0156*	0.5956	*0.0136*	1.0000	1.0089	0.4852	− 1.3705	0.1370	*1.6324*	0.4615	− 1.3827	0.1479	*1.6181*	*0.0052*	*2.2373*
*Magi2*	0.2062	*0.0802*	*0.0467*	0.9441	1.1186	0.1484	*− 1.5570*	0.9976	1.0386	*0.0475*	*− 1.7416*	0.9826	− 1.0770	0.1031	*1.6170*
*Parp3*	*0.0694*	0.4886	*0.0026*	0.7494	1.1354	0.2804	− 1.2612	0.2761	1.2628	*0.0398*	− 1.4320	0.8355	1.1122	*0.0053*	*1.5927*
*Cass4*	*0.0414*	0.7084	*0.0023*	0.8262	1.1739	0.3046	− 1.3943	0.1772	1.4813	*0.0614*	*− 1.6368*	0.6049	1.2618	*0.0032*	*2.0654*

Our list of previously published blood transcriptome biomarkers of AD is shown in Table 4 [7–15]. The *Trem1* mRNA levels tended to increase with age only in the control mouse blood. The *Trem1* mRNA level showed significant increases with age in the control mouse brain and was significantly lower in the AD mouse brain than in the control mouse brain at 52 w.o.a. The *Trem2* mRNA level tended to increase with age in the control mouse blood and was lower in the AD mouse blood than in the control mouse blood at 52 w.o.a. The *Trem2* mRNA level significantly increased with age in the AD mouse brain and was significantly higher in the AD mouse brain than in the control mouse brain. The *Mef2c* mRNA level showed significant increases with age in the control mouse brain. The *Tomm40* mRNA level tended to decrease with age in the blood of both the AD and control mice. The *Tomm40* mRNA level significantly decreased with age in the AD mouse brain and was significantly lower in the AD mouse brain than in the control mouse brain at 52 w.o.a. The *Pink1* mRNA level was significantly higher in the AD mouse blood than in the control mouse blood at both 12 and 52 w.o.a. The *Pink1* mRNA level tended to increase with age and was usually lower in the AD mouse brain. The *Apoe* mRNA level significantly increased with age in the control mouse blood and was significantly lower in the AD mouse blood than in the control mouse blood at both 12 and 52 w.o.a. The *Apoe* mRNA level showed significant increases with age in the AD mouse brain and was significantly higher in the AD mouse brain than in the control mouse brain at 52 w.o.a. The *Inpp5d* mRNA level was significantly higher in the AD mouse brain than in the control mouse brain at both 12 and 52 w.o.a. The *Snca* mRNA level was significantly higher in the AD mouse blood than in the control mouse blood at both 12 and 52 w.o.a.

**Table 4:** List of our previously identified candidate blood transcriptome biomarkers of AD (C12, control mice at 12 w.o.a.; C52, control mice at 52 w.o.a.; AD12, AD mice at 12 w.o.a.; AD52, AD mice at 52 w.o.a.; bld, blood; hip, hippocampus; FC, fold change; w.o.a., weeks of age). The significantly altered genes (two-way ANOVA: *P* < 0.1, fold change ≥ 1.5 or ≤ − 1.5, Tukey’s HSD: *P* < 0.05) are indicated in italics

**Gene symbol**	**p (Mouse-Age)**	**p (Mouse)**	**p (Age)**	**C12 bld vs. C52 bld**		**C12 bld vs. AD12 bld**		**C12 bld vs. AD52 bld**		**C52 bld vs. AD12 bld**		**C52 bld vs. AD52 bld**		**AD12 bld vs. AD52 bld**	
				**p**	**FC**	**p**	**FC**	**p**	**FC**	**p**	**FC**	**p**	**FC**	**p**	**FC**
*Ghrl*	*0.0113*	0.1613	0.7192	0.1556	1.3857	0.8054	1.1445	0.8714	− 1.1209	0.5865	− 1.2108	*0.0317*	*− 1.5533*	0.3633	− 1.2829
*Ghsr*					1.1210		1.0934		1.0484		− 1.0252		− 1.0693		− 1.0430
*Mboat4*					− 1.1921		1.0079		1.3297		1.2015		*1.5851*		1.3193
*Trem1*	0.5174	0.8484	0.2043	0.5204	*1.6086*	0.9877	1.1191	0.8618	1.3087	0.7189	− 1.4374	0.9313	− 1.2291	0.9681	1.1695
*Trem2*	*0.0463*	*0.0952*	0.4028	0.1860	*1.5407*	0.9942	1.0543	0.9248	− 1.1379	0.2848	− 1.4614	*0.0541*	*− 1.7532*	0.8183	− 1.1996
*Mef2c*					− 1.4080		1.2968		*− 1.5502*		*1.8259*		− 1.1010		*− 2.0103*
*Tomm40*	0.6994	0.3807	*0.0262*	0.2368	− 1.4794	0.8020	− 1.2012	0.1249	*− 1.5893*	0.7338	1.2317	0.9845	− 1.0743	0.5198	− 1.3231
*Pink1*	0.8599	*0.0000*	*0.0065*	0.1467	1.3464	*0.0014*	*1.7626*	*0.0002*	*2.2938*	0.2134	1.3091	*0.0027*	*1.7037*	0.2298	1.3014
*Apoe*	*0.0006*	*0.0000*	*0.0559*	*0.0017*	*2.4561*	*0.0468*	*− 1.8234*	*0.0020*	*− 2.4246*	*0.0002*	*− 4.4786*	*0.0002*	*− 5.9553*	0.5646	− 1.3297
*Inpp5d*	*0.0945*	0.2560	0.6740	0.7928	1.1030	0.9774	1.0438	0.6803	− 1.1264	0.9538	− 1.0567	0.1966	− 1.2425	0.4370	− 1.1758
*Snca*	*0.0664*	*0.0000*	0.1774	0.1155	*1.6118*	*0.0002*	*2.9087*	*0.0004*	*2.6949*	*0.0358*	*1.8047*	*0.0805*	*1.6720*	0.9821	− 1.0793
*Abca7*	0.6452	0.1091	0.9318	0.9795	1.0718	0.4514	1.3053	0.6129	1.2446	0.6871	1.2179	0.8345	1.1612	0.9932	− 1.0488
*Slc6a4*					− 1.3188		− 1.0864		*1.5138*		1.2139		*1.9963*		*1.6445*
**Gene symbol**	**p (Mouse-Age)**	**p (Mouse)**	**p (Age)**	**C12 Hip vs. C52 Hip**		**C12 Hip vs. AD12 Hip**		**C12 Hip vs. AD52 Hip**		**C52 Hip vs. AD12 Hip**		**C52 Hip vs. AD52 Hip**		**AD12 Hip vs. AD52 Hip**	
				**p**	**FC**	**p**	**FC**	**p**	**FC**	**p**	**FC**	**p**	**FC**	**p**	**FC**
*Ghrl*	0.4572	0.2064	0.7307	0.8634	− 1.0492	0.4815	− 1.0934	0.6561	− 1.0742	0.9080	− 1.0421	0.9808	− 1.0238	0.9916	1.0179
*Ghsr*	0.5031	0.3105	0.6870	0.8684	1.1799	0.9944	− 1.0555	0.9706	− 1.1001	0.7403	− 1.2454	0.6258	− 1.2980	0.9975	− 1.0422
*Mboat4*					− 1.0737		*− 2.0604*		*− 2.0574*		*− 1.9189*		− 1.9161		1.0015
*Trem1*	*0.0250*	*0.0003*	*0.0616*	*0.0241*	1.1178	0.6222	− 1.0454	0.4432	− 1.0568	*0.0012*	− 1.1684	*0.0006*	− 1.1812	0.9907	− 1.0109
*Trem2*	*0.0129*	*0.0000*	*0.0000*	0.2474	1.1068	0.4250	1.0856	*0.0002*	1.4672	0.9834	− 1.0195	*0.0002*	1.3256	*0.0002*	1.3514
*Mef2c*	*0.0833*	0.6316	*0.0440*	*0.0468*	*1.5712*	0.3882	1.3020	0.2791	1.3501	0.6633	− 1.2068	0.7908	− 1.1638	0.9961	1.0369
*Tomm40*	*0.0002*	*0.0000*	*0.0028*	0.8890	1.0126	0.3673	− 1.0293	*0.0002*	− 1.1302	0.1065	− 1.0423	*0.0002*	− 1.1445	*0.0002*	− 1.0980
*Pink1*	0.5597	*0.0254*	*0.0231*	0.1728	1.0658	0.5997	− 1.0385	1.0000	1.0009	*0.0113*	− 1.1068	0.1820	− 1.0649	0.5819	1.0394
*Apoe*	*0.0005*	*0.0006*	*0.0262*	0.6810	− 1.0345	1.0000	− 1.0012	*0.0009*	1.1425	0.7046	1.0332	*0.0002*	1.1818	*0.0008*	1.1438
*Inpp5d*	0.6691	*0.0000*	0.1260	0.4975	1.0662	*0.0047*	1.1832	*0.0007*	1.2272	0.1209	1.1097	*0.0208*	1.1510	0.8493	1.0372
*Snca*					1.2665		*1.8059*		*1.5751*		1.4259		1.2437		− 1.1465
*Abca7*	0.3479	*0.0374*	0.6671	0.9827	− 1.0371	0.8200	1.0900	0.2710	1.2013	0.6086	1.1304	0.1424	1.2458	0.7605	1.1021
*Slc6a4*					*1.6336*		*1.5084*		*1.9392*		− 1.0830		1.1871		1.2856

### Real-Time PCR Validation of DEGs in Microarray Data

**Fig. 1:**
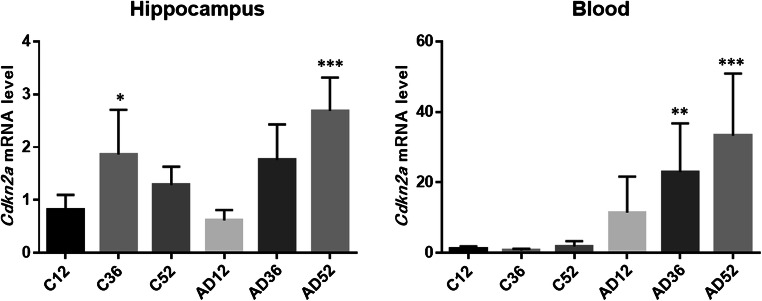
Validation of *Cdkn2a* by qPCR. The *Cdkn2a* mRNA level in both the blood and hippocampus showed significant increases with age only in AD mice. The data (*n* = 8) are shown as the means ± SEMs. *, **, and *** indicate *P* < 0.05, 0.01, and 0.001, respectively. * vs. the expression in C12 (control mice at 12 w.o.a.), as determined by one-way ANOVA with Dunnett’s multiple comparisons test. C12, control mice at 12 w.o.a.; C36, control mice at 36 w.o.a; C52, control mice at 52 w.o.a.; AD12, AD mice at 12 w.o.a.; AD36, AD mice at 36 w.o.a.; AD52, AD mice at 52 w.o.a

To verify the reliability of the microarray data, we selected *Cdkn2a* for qPCR experiments because this gene exhibited the greatest changes among the five candidate genes and is well known as an aging- and cellular senescence-associated gene [23, 24]. This analysis aimed to not only validate the correlations between the microarray and real-time PCR data but also confirm the detection of well-known changes. The *Cdkn2a* mRNA level in the blood and hippocampus significantly increased with age only in the AD mice (Fig. 1). The data (*n* = 8 each group) were assessed by one-way ANOVA with Dunnett’s multiple comparisons test. The significance was set to *P* < 0.05. The results further validated the results of the microarray data.

### Validation of DEGs in the Human Postmortem Hippocampus and APP_PS1 Mouse Hippocampus

A substantial number of candidate genes in the human postmortem hippocampus (Supplementary Figure 2) and APP_PS1 hippocampus (Supplementary Figure 3) showed similar changes to our microarray data. In particular, several genes (APOBEC3, PARP3, TREM2, TOMM40, APOE, and INPP5D) changed significantly in the same direction in both human AD and AD model mice, and the results further validated the results of the microarray data.

### Discriminant Analyses

Discriminant analysis of the AD52 bld and C52 bld with the variables Cdkn2a, Apobec3, Magi2, Parp3, Cass4, Trem1, Trem2, Tomm40, Pink1, Apoe, Inpp5d, and Snca revealed that the combination of 3 genes (Apoe, Cass4, Cdkn2a) resulted in the best prediction (Wilks lambda = 0.170, *P* < 0.001). The discrimination score (*D*) was calculated for each sample as follows:

*D* = −0.697 × Apoe + 0.635 × Cass4 + 0.518 × Cdkn2a − 0.57749

The analysis showed a sensitivity and specificity of 100.0% and 100.0%, respectively (Supplementary Figure 4 A).

Discriminant analysis of the AD52 Hip and C52 Hip was also performed with the same gene combination (Wilks lambda = 0.190, *P* < 0.001). The discrimination score was calculated for each sample as follows:

*D* = 0.735 × Apoe − 0.113 × Cdkn2a + 0.091 × Cass4–0.742122

The analysis established a sensitivity and specificity of 100.0% and 100.0%, respectively (Supplementary Figure 4 B).

To validate biomarkers in the blood of patients with Alzheimer’s disease, we used the expression data from the Gene Expression Omnibus database (GSE97760). [25] Discriminant analysis of the AD patients (*N* = 9) and healthy subjects (*N* = 10) was conducted with the same gene combination (APOE, CASS4, CDKN2A) (Wilks lambda = 0.577, *P* = 0.036). The discrimination score was calculated for each sample as follows:

*D* = −0.742 × APOE × 0.696 × CDKN2A − 0.473 × CASS4 + 860.715

The analysis demonstrated a sensitivity and specificity of 77.8% and 80.0%, respectively (Supplementary Figure 4 C).

## Discussion

The main purposes of the study were to identify novel blood transcriptome biomarkers of AD and to validate our previous biomarkers. Transcriptome biomarkers that exhibit significant changes in both the blood and hippocampus might have potential as minimally invasive and inexpensive markers for the diagnosis and early detection of developing AD. The lack of consistent correlations among different tissues indicates the difficulty in identifying blood biomarkers that exhibit positive correlations between the blood and hippocampus only in AD mice (Supplementary Figure 1). Because several genes (APOBEC3, PARP3, TREM2, TOMM40, APOE, and INPP5D) changed significantly in the same direction in multiple datasets from both human AD samples and mouse AD models, these genes may be good candidates for blood biomarkers for AD (Supplementary Figure 2 and Supplementary Figure 3). According to discriminant analyses, the combination of 3 genes (Apoe, Cass4, Cdkn2a) was the most useful to discriminate not only the blood and the hippocampus of AD mice but also the blood of AD patients (Supplementary Figure 4).

Interestingly, “immunological disease,” “organismal injury and abnormalities,” “cell-to-cell signaling and interaction,” “cell death and survival,” “cellular growth and proliferation,” and “hematological system development and function” were the most common diseases and biofunctions found in the comparison between the blood and hippocampus. Furthermore, the DEGs that were uniquely found in the AD mouse hippocampus were involved in “altered T cell and B cell signaling in rheumatoid arthritis,” “communication between innate and adaptive immune cells,” and “immune cell trafficking.” Consistently, the proinflammatory activity of microglia is related to behavioral alterations in AD patients and experimental models of the disease [26]. DEGs that were only found in the AD mouse blood were involved in “primary immunodeficiency signaling,” “inflammatory response,” and “lymphoid tissue structure and development.” These results provide further evidence showing the important roles of the immune system in both the brain and blood in the pathogenesis of AD [27, 28].

The expression of five gene transcripts (*Cdkn2a*, *Apobec3*, *Magi2*, *Parp3*, and *Cass4*) significantly increased with age, and their expression in the blood was correlated to that in the hippocampus only in AD mice (Table 3). CDKN2A is a well-known molecular player in cellular senescence, cell proliferation, survival, adhesion, and apoptosis [23, 24]. An increased level of *Cdkn2a* is present in both brain and blood cells from APP/PS1 mice [29]. Interestingly, linkage and association studies have linked the *CDKN2A* locus (9p21.3) to late-onset AD families [30]. Human APOBEC3 plays important roles in intracellular defense against viral infection and cancer development by generating DNA mutations [31]. However, no previous studies have found associations between APOBEC3 and AD. MAGI2 regulates apoptosis, cytoskeletal reorganization, and glomerular development and is broadly expressed in the brain, thyroid, and 16 other tissues, including blood [32]. MAGI2 is a candidate gene associated with multiple phenotypes, as demonstrated by Alzheimer’s Disease Neuroimaging Initiative genetic studies [33]. The protein encoded by PARP3 belongs to the PARP family, and the members of this family modify nuclear proteins via poly-ADP-ribosylation, which is required for DNA repair, the regulation of apoptosis, and the maintenance of genomic stability [34]. Although PARP3 is a target in cancer therapy [35], no previous study has revealed an association with AD. CASS4 is a well-known candidate gene of AD [36] and has recently been studied in the context of immune system function and the pathogenesis of developmental and autoimmune disorders, including Crohn’s disease, cancer, and other diseases [37].

Our previously identified candidate genes also showed significant changes in both the blood and brain (Table 4). The expression of *Trem1* and *Trem2* increased in the control mouse blood with age. Consistent with our previous studies, which showed higher *TREM1* and *TREM2* mRNA levels in the human AD blood [8–10], the levels of these mRNAs in the control mouse blood tended to increase with age. These results suggest that higher *TREM1* and *TREM2* mRNA levels in human AD blood might reflect abnormal aging rather than pathophysiological changes in AD. However, the identification of significant changes in the *Trem1* and *Trem2* mRNA levels in the mouse brain indicated their important roles in the development of AD. Consistent with our previous study [7], decreased *Tomm40* and increased *Pink1* mRNA levels in mouse blood were observed in the current study. Because both *TOMM40* and *PINK1* play important roles in mitochondrial function, these results provide further evidence of mitochondrial dysfunction in AD [38, 39]. The *Apoe* mRNA level significantly increased with age in only the control mouse blood and was significantly lower in the AD mouse blood than in the control mouse blood. Although our previous study revealed that the *APOE* mRNA level was not changed in human AD blood [7], a recent study showed that the *APOE* mRNA level is significantly increased in human AD blood [40]. Consistent with our previous studies [41, 42], the *Snca* mRNA levels significantly increased with age in the control mouse blood and were significantly higher in the AD mouse blood than in the control mouse blood. These results indicate that the *APOE* and *SNCA* mRNA levels in human blood might be candidate markers for aging and AD.

This study has several limitations. First, our results should be validated using liquid biopsies of AD patients obtained from a certificated biobank. Second, because male 3xTg AD mice exhibit progressive behavioral and pathological changes at 36 and 52 w.o.a. [14, 15], we did not check these changes prior to tissue collection. However, the investigator who donated the 3xTg AD mice communicated that in contrast to the initial observations, male transgenic mice might not exhibit the originally described phenotypic traits. Thus, we should have checked their symptoms and should also examine whether female mice also exhibit the same changes. The positive correlations in the expression of all tested genes found among the subgroups from the same group of mice suggest that the male 3xTg AD mice in this study did not lose their pathological phenotype (Supplementary Figure 1). Third, although we identified five genes that showed significant increases in expression with age and showed correlations in expression between the blood and hippocampus only in AD mice, we only confirmed the changes in the expression of one gene (*Cdkn2a*) due to sample shortage. The changes in the other four genes should be confirmed using both AD female mouse samples and human AD blood samples in future studies. Further analyses of the microarray data using complex algorithms, such as machine learning and deep learning, will be conducted in the future.

## Conclusion

The results from our comprehensive analysis using brain and blood samples from transgenic mice provide insights into putative transcriptomic biomarkers of AD. The results suggest that the expression of immune-associated genes exhibits changes in not only the blood but also the hippocampus. Because five genes (*Cdkn2a*, *Apobec3*, *Magi2*, *Parp3*, and *Cass4*) were detected as novel candidate transcriptomic biomarkers of AD, these results need to be validated through further studies using human blood and brain samples. The changes in the expression of some of our candidate genes, such as *Trem2*, *Tomm40*, and *Snca*, in the mouse blood and brain were similar to those in the human AD blood. The combination of 3 genes (Apoe, Cass4, Cdkn2a) may be the most useful AD biomarker in the blood. This study provided further evidence showing that blood transcriptome markers have potential use as biomarkers of AD.

## Electronic supplementary material

Supplementary Figure 1 Correlations among the expression of all tested genes among all the groups. Positive correlations (red) were found among the same tissues of the mice belonging to the same group, such as between the AD12 and AD52 hip samples, whereas negative correlations (blue) were detected among the same tissues of the mice belonging to different groups, such as between the AD hip samples and the C hip samples. No consistent correlations (yellow) were observed among different tissues, such as between the AD blood and hip samples. (TIF 481 kb) (PNG 5360 kb)

High resolution image (TIF 481 kb)

Supplementary Figure 2 Validation analyses of candidate gene expression in human postmortem hippocampal samples from the expression data of the Gene Expression Omnibus database (GSE48350). Young male controls (*N*=9, mean age: 30.6 ± 11.0), old male controls (*N*=10, 85.8 ± 6.8) and male patients with Alzheimer’s disease (*N*=9, 84.1 ± 6.9) were compared. Statistical significance was determined by one-way ANOVA with Tukey’s multiple comparisons test (*P*<0.05). (TIF 103 kb) (PNG 5360 kb)

High resolution image (TIF 103 kb)

Supplementary Figure 3 Validation analyses of candidate gene expression in APP_PS1 hippocampal samples from the expression data of the Gene Expression Omnibus database (GSE111737). Wild-type male mice (*n*=6, 8 months old) and APP_PS1 male mice (*n*=7, 8 months old) were compared. Statistical significance was determined by t-test (P<0.05). (TIF 360 kb) (PNG 5360 kb)

High resolution image (TIF 360 kb)

Supplementary Figure 4 A The distribution of Discrimination scores (○: C52 bld, ●: AD52 bld) with a sensitivity and specificity of 100.0% and 100.0%, respectively. B. The distribution of Discrimination scores (○: C52 Hip, ●: AD52 Hip) with a sensitivity and specificity of 100.0% and 100.0%, respectively. C. The distribution of Discrimination scores (○: the blood of healthy subjects (N=10), ●: the blood of AD patients (N=9)) with a sensitivity and specificity of 77.8% and 80.0%, respectively. (TIF 91 kb) (PNG 5360 kb)

High resolution image (TIF 91 kb)

ESM 5 (XLSX 18 kb)

## Data Availability

All microarray data were deposited in the GEO database (accession number GSE144459).
