# Supplementary figures and images for: Identifying Blood Transcriptome Biomarkers of Alzheimer’s Disease Using Transgenic Mice
Source: Mol Neurobiol. 2020 Aug 20;57(12):4941–51. doi: 10.1007/s12035-020-02058-2 (PMC7541363; doi:10.1007/s12035-020-02058-2)

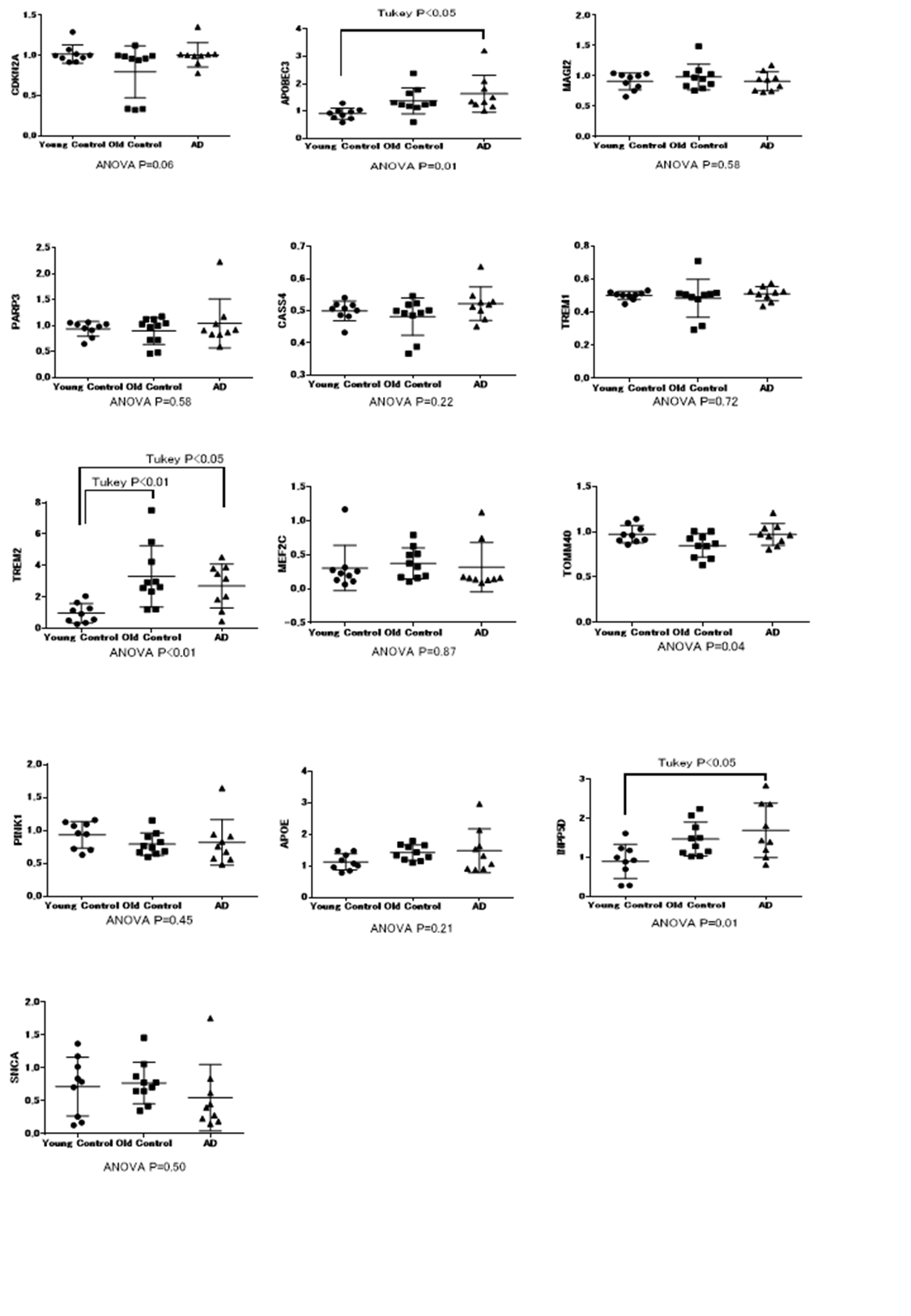

Supplement: Supplementary file 1 — Correlations among the expression of all tested genes among all the groups. Positive correlations (red) were found among the same tissues of the mice belonging to the same group, such as between the AD12 and AD52 hip samples, whereas negative correlations (blue) were detected among the same tissues of the mice belonging to different groups, such as between the AD hip samples and the C hip samples. No consistent correlations (yellow) were observed among different tissues, such as between the AD blood and hip samples. (TIF 481 kb) (PNG 5360 kb) [file 12035_2020_2058_Fig2_ESM.png]

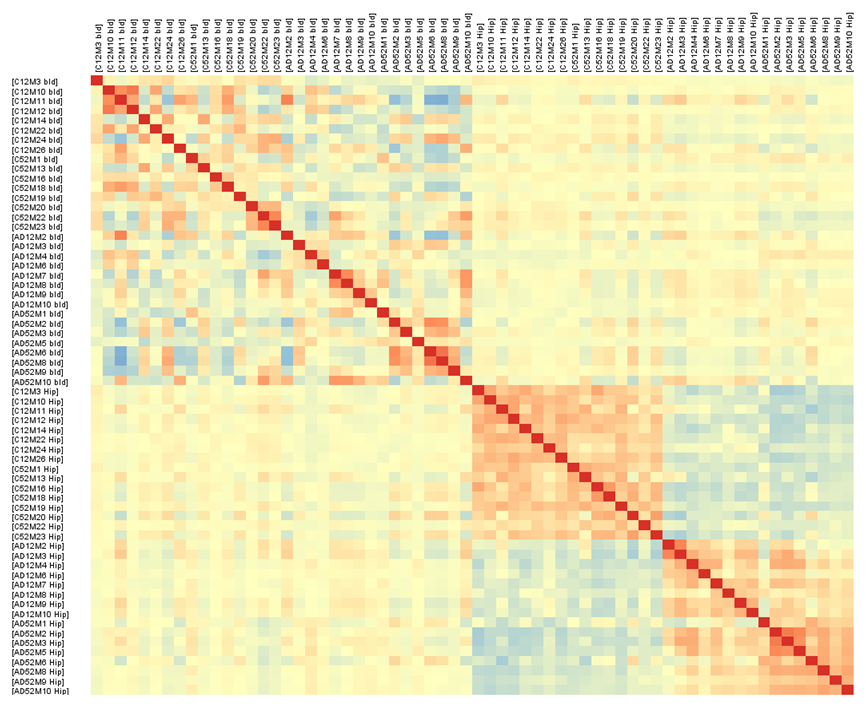

Supplement: Supplementary file 2 — High resolution image (TIF 481 kb) [file 12035_2020_2058_MOESM1_ESM.tif]

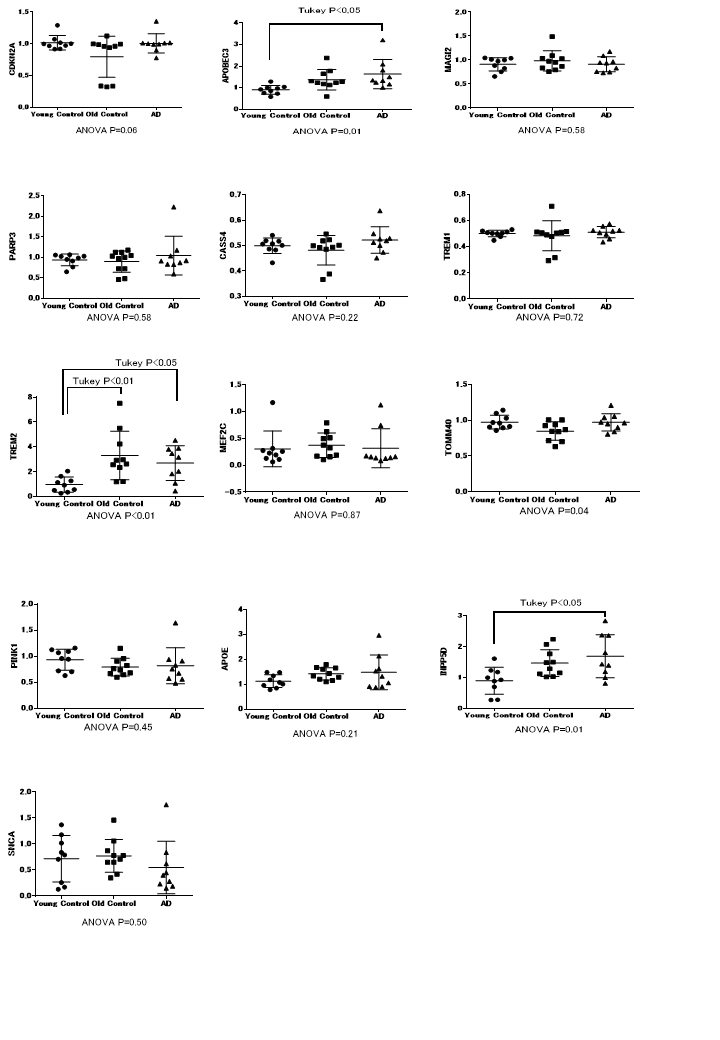

Supplement: Supplementary file 4 — High resolution image (TIF 103 kb) [file 12035_2020_2058_MOESM2_ESM.tif]

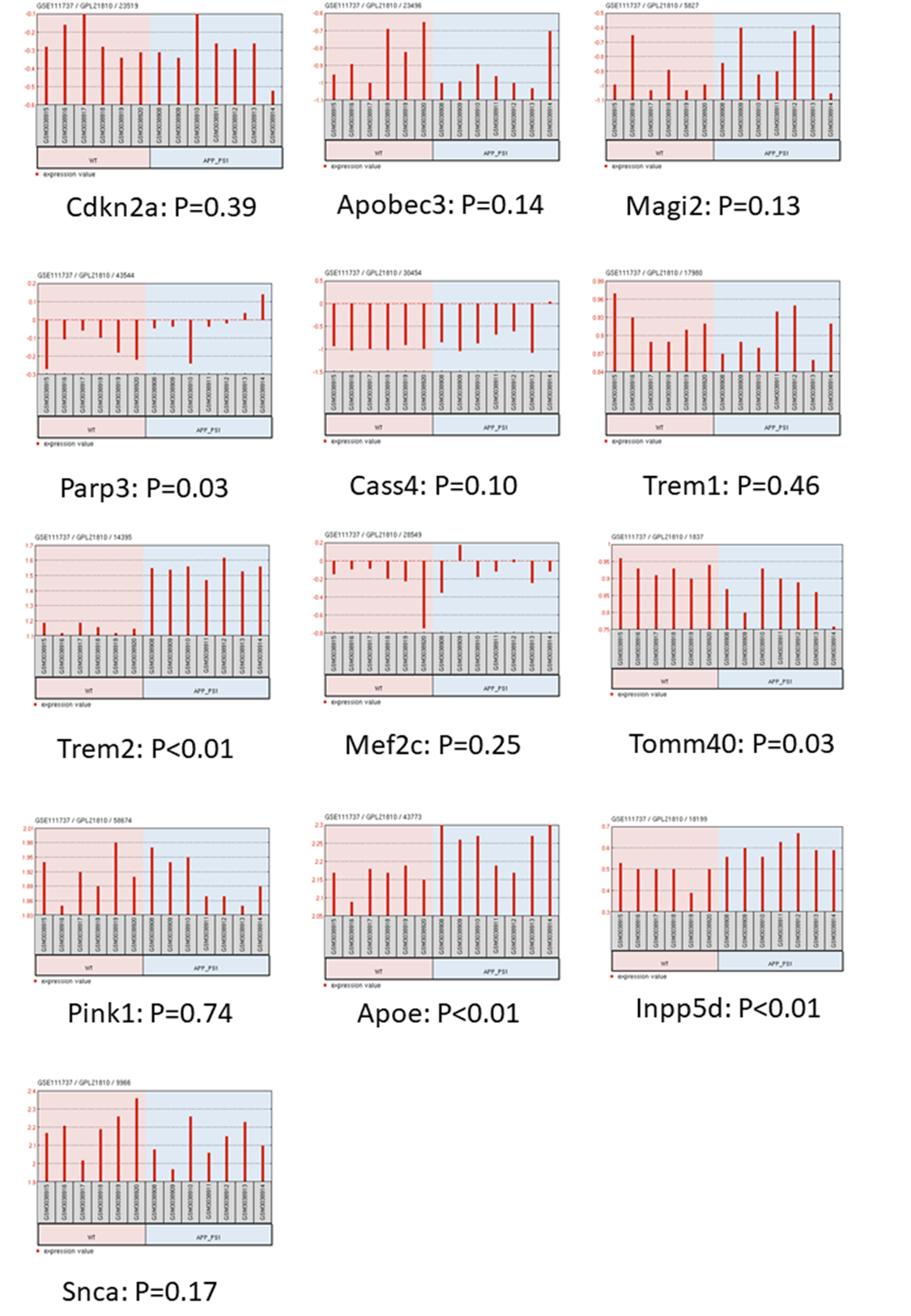

Supplement: Supplementary file 5 — Validation analyses of candidate gene expression in APP_PS1 hippocampal samples from the expression data of the Gene Expression Omnibus database (GSE111737). Wild-type male mice (n=6, 8 months old) and APP_PS1 male mice (n=7, 8 months old) were compared. Statistical significance was determined by t-test (P<0.05). (TIF 360 kb) (PNG 5360 kb) [file 12035_2020_2058_Fig4_ESM.png]

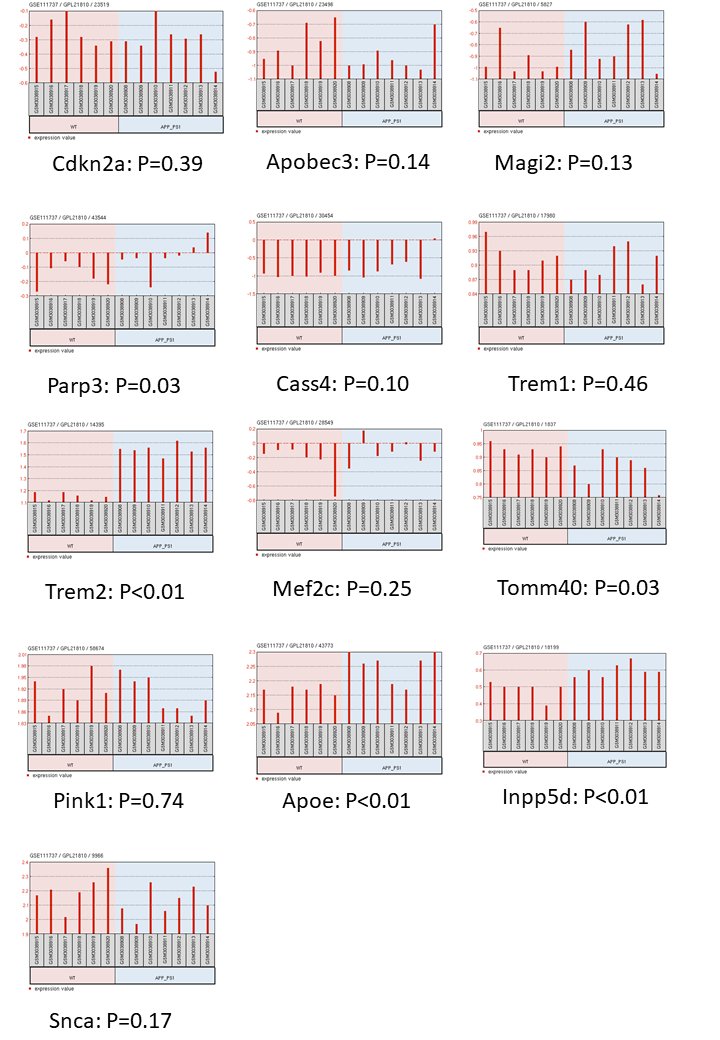

Supplement: Supplementary file 6 — High resolution image (TIF 360 kb) [file 12035_2020_2058_MOESM3_ESM.tif]

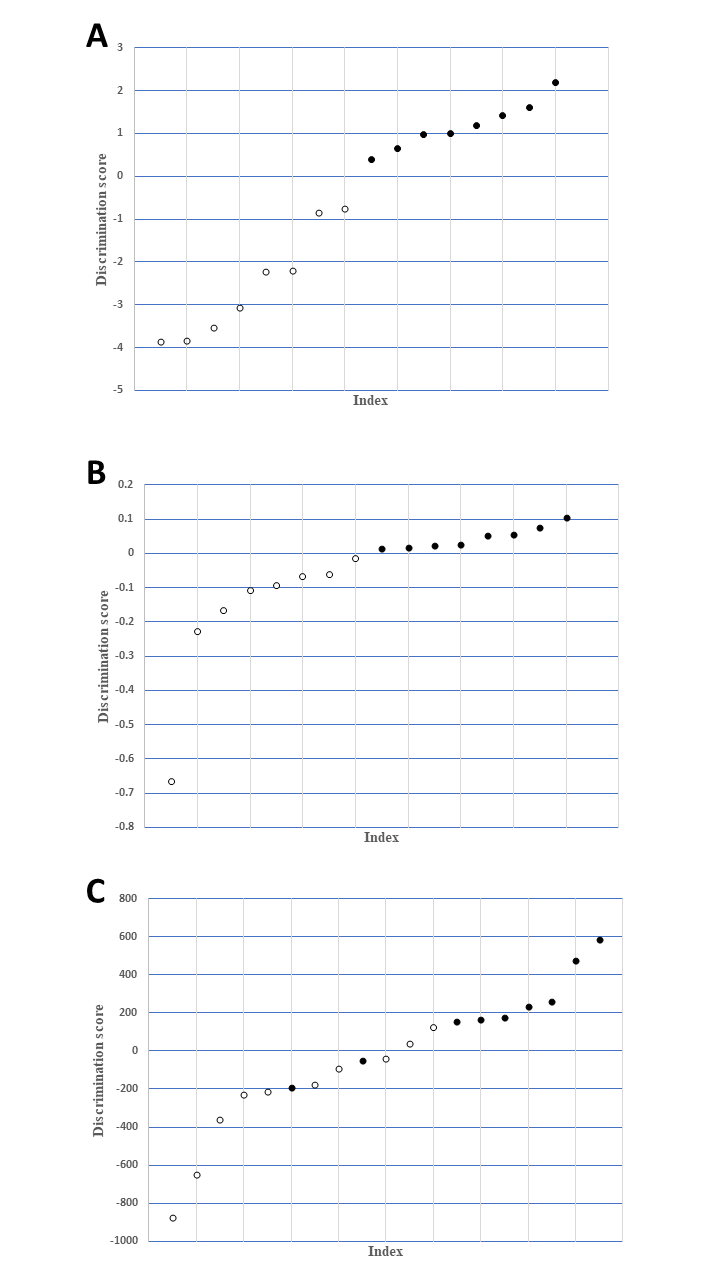

Supplement: Supplementary file 8 — High resolution image (TIF 91 kb) [file 12035_2020_2058_MOESM4_ESM.tif]
